# A Comparison of Physiological Homeostasis and Product Quality Between Recirculating Water and Earthen Pond Culture of *Litopenaeus vannamei*

**DOI:** 10.3390/biology15201823

**Published:** 2026-10-12

**Authors:** Senyue Liu, Yu He, Quan Gong, Yang Feng, Shuai Liu, Zhipeng Huang, Yuanliang Duan, Dan Song, Pengcheng Li, Jian Zhou, Yongqiang Deng

**Affiliations:** 1 Fisheries Research Institute, Sichuan Academy of Agricultural Sciences (Sichuan Fisheries Research Institute), Chengdu 611731, China; 2 Aquatic Health and Intelligent Aquaculture Key Laboratory of Sichuan Province, Chengdu 611130, China; 3 Department of Aquaculture, Fisheries College, Sichuan Agricultural University, Chengdu 611130, China

**Keywords:** recirculating aquaculture system, earthen pond culture, physiological homeostasis, tissue ammonia, immune suppression, *Litopenaeus vannamei*

## Abstract

Water quality deterioration during the late culture phase remains a key limiting factor for intensive *Litopenaeus vannamei* farming. This study compared traditional open earthen ponds and recirculating aquaculture systems (RAS) to investigate differences in physiological health, tissue structure, and muscle quality of cultured shrimp between the two culture modes. Using coefficient of variation (CV) as a quantitative metric, we observed that water quality parameters in the RAS group exhibited smaller fluctuation amplitudes, with nitrogenous metabolite concentrations and TCBS-culturable *Vibrio*-like bacteria loads notably lower than those in open earthen ponds. Shrimp reared in RAS achieved better overall growth performance. In contrast, shrimp in the pond system showed systemic ammonia accumulation across multiple tissues, accompanied by gill lamellar blockage, respiratory epithelial shedding, and disrupted mitochondrial cristae in the hepatopancreas. Histochemical assays further revealed marked glycogen depletion in the hepatopancreas of pond-reared shrimp, along with a “low-protein, high-moisture” muscle profile characterized by porous myofibrillar architecture. Although oxidative stress markers were elevated in the Pond group, plasma lysozyme activity was significantly suppressed. These findings suggest that the superior growth performance and muscle quality observed in the RAS system may be partly associated with lower homeostatic and stress repair costs, which arise from overall differences in rearing conditions between the two culture modes. The present study provides supporting histological and physiological evidence for optimizing shrimp culture strategies and upgrading production models.

## Simple Summary

Pacific white shrimp is one of the most widely farmed shrimp species globally. In traditional open-pond farming, water quality often grows unstable in the late rearing stage, harming shrimp health, slowing growth, and degrading meat quality. This study compared traditional open ponds with closed recirculating farming systems to examine the physiological and quality differences in shrimp between the two culture systems. The recirculating system maintained far more controlled water and lower harmful bacteria levels. Shrimp raised there grew larger, with healthier tissues, stronger disease resistance, and more nutritious meat. By contrast, fluctuating water quality in traditional ponds caused harmful substance accumulation, tissue damage, and weakened immunity in shrimp. More controlled rearing conditions allow shrimp to allocate more energy to growth rather than stress coping. These findings help improve farming practices for higher-quality aquatic products and more sustainable aquaculture.

## 1. Introduction

The Pacific white shrimp (*Litopenaeus vannamei*, *L. vannamei*) has emerged as one of the most widely distributed and highest-valued crustacean species in global intensive aquaculture, attributed to its exceptional growth performance and euryhaline adaptability [1]. As stocking densities continue to rise, however, traditional open-pond systems face growing pressure on environmental carrying capacity and biosecurity [2]. In these open systems, roughly 70% of total ammonia nitrogen (TAN) originates from microbial breakdown of high-protein feed residues and animal metabolic waste [3]. With limited buffering capacity against climatic fluctuations and a lack of active environmental control, water conditions in ponds tend to become highly unstable as culture progresses. This instability is most pronounced during the late culture stage: as biomass peaks, waste accumulation drives sustained increases in TAN and nitrite, coupled with sharp swings in water temperature, dissolved oxygen, and pH [4]. Together, these disturbances gradually erode the physiological defense capacity of cultured shrimp.

Recirculating aquaculture systems (RAS) address these bottlenecks by integrating mechanical filtration with biological nitrification [5,6]. The production advantages of closed systems over open ponds, including improved growth, reduced FCR, and more controlled nitrogen levels, have been widely documented across indoor RAS [7,8], biofloc tank systems [9,10], and super-intensive regulated ponds [11]. However, most studies have focused on macro-level production metrics, while the physiological and histological underpinnings remain poorly understood across multiple biological scales.

Toxicological impacts of environmental stress exhibit distinct tissue specificity. As the primary interface for gas exchange and osmoregulation, gills form the first physical barrier against exogenous toxins, and environmental disturbance is known to trigger gill dysfunction and impair active ion transport [12,13]. The hepatopancreas acts as the metabolic and immune-regulatory hub of shrimp, governing energy allocation, detoxification, and oxidative defense [14,15]. When environmental fluctuations exceed physiological thresholds, pathological accumulation of tissue ammonia occurs, stimulating excessive reactive oxygen species (ROS) production, disrupting redox balance, and inducing irreversible subcellular damage [16].

The late culture stage represents a critical window for comparing physiological responses under different culture modes, as pathological damage reaches its cumulative peak alongside maximum environmental load and animal biomass. Building on the research gaps outlined above, we proposed the core scientific hypothesis that the culture mode (RAS vs. open earthen ponds) exerts significant effects on the growth performance, tissue structural integrity and muscle nutritional quality of *L. vannamei*, and that these phenotypic variations may be linked to differences in tissue ammonia burden, oxidative status, and metabolic energy allocation. To test this hypothesis, we characterized water quality dynamics, quantified multi-tissue ammonia concentrations, examined histopathological features, and assessed oxidative and immune responses in shrimp from both systems. We further aimed to clarify the cyto-histological mechanisms driving differential growth performance, with a focus on metabolic allocation patterns. These findings provide theoretical support for optimizing shrimp farming practices and health management strategies.

## 2. Materials and Methods

### 2.1. Experimental Site and System Design

**Figure 1:**
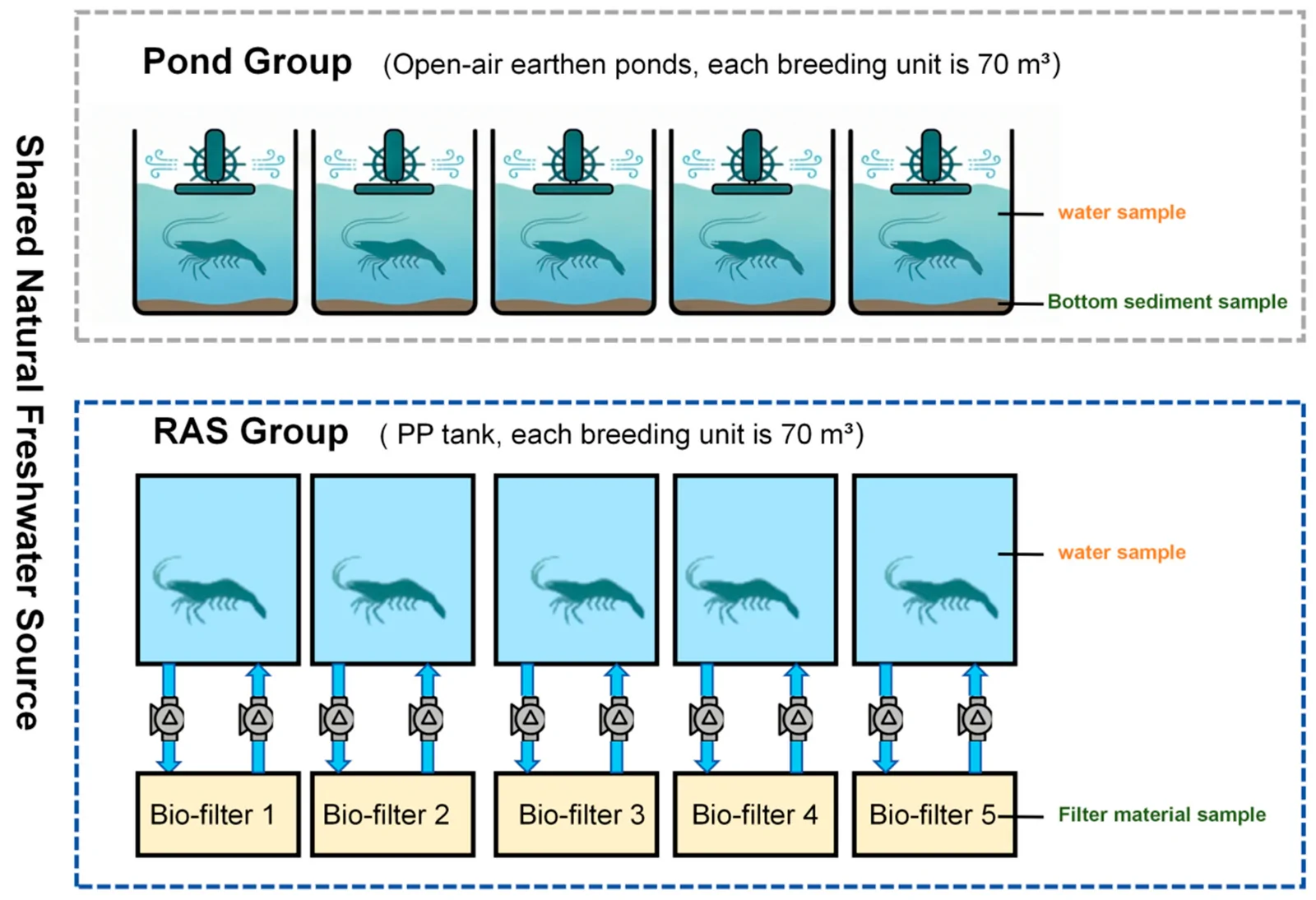
Design model diagram of the experimental site and breeding system.

This experiment was conducted at the Fisheries Research Institute, Sichuan Academy of Agricultural Sciences in Chengdu, China. Two culture modes were established as experimental treatments (Figure 1): traditional open ponds and RAS. Each treatment included five independent culture units (*n* = 5), with an effective water volume of 70 m^3^ per unit. Both groups received the same batch of fully aerated natural freshwater with a salinity below 0.5‰.

The Pond group adopted conventional open-air earthen ponds. Water quality in these ponds was maintained via natural ecological self-purification, and impeller aerators were equipped to support dissolved oxygen levels. The RAS group was constructed through in situ modification within the original pond farming area to ensure consistent geographic and climatic background conditions. Each culture tank was fabricated from double-layer polypropylene with an integrated thermal insulation layer. Each RAS unit achieved a dedicated closed loop circulation through pipelines connected to an independent biochemical compartment. These compartments were retrofitted from two existing standard ponds and divided into five separate units, matching the number of biological replicates in the Pond group. The system integrated mechanical drum filters, biofilters, circulating pumps, and microporous aeration to achieve water quality control through efficient nitrification and physical filtration.

This in situ modification ensured that both groups shared identical ambient conditions and water sources, ruling out confounding effects caused by macro-environmental background differences.

### 2.2. System Management and Activation

Prior to shrimp stocking, a system activation and pretreatment phase was performed to establish stable initial water conditions for both culture modes.

For the RAS group, a 21-day biofilm pre-cultivation was conducted to achieve a stable nitrification steady state. Analytical-grade ammonium chloride (NH_4_Cl, aladdin, Shanghai, China) was prepared as a concentrated stock solution and continuously dosed into the biofilter to provide a nitrogen substrate for microbial colonization. A commercial composite nitrifying bacteria preparation (Q/371701NWH 007 2023, Nuoweihai, Heze, China) was inoculated to accelerate biofilm formation on the filter media. Nitrogen parameters were monitored daily to track the progression of nitrification performance. The biofilm was considered mature when the system maintained total ammonia nitrogen ≤ 0.3 mg/L and nitrite nitrogen ≤ 0.1 mg/L for three consecutive days. This maturity signaled that the system possessed reliable nitrogen processing capacity for the formal culture stage.

In parallel, the Pond group received circulating aeration pretreatment combined with physical and ecological regulation. This step was implemented to ensure initial water quality parameters and dissolved oxygen levels were consistent with those in the RAS group at the onset of stocking.

### 2.3. Experimental Animals and Management

Healthy post-larval stage 4 (PL4) *L. vannamei* (0.82 ± 0.05 cm, 0.0042 ± 0.0006 g) pre-acclimated to freshwater conditions were purchased from a commercial hatchery in Chengdu, Sichuan, China. The salinity of the shrimp at delivery was below 0.5‰, consistent with the experimental culture water.

Prior to the formal experiment, all shrimp underwent a 7-day indoor acclimation period in polypropylene tanks supplied with the same aerated freshwater used for the culture systems. During acclimation, shrimp were fed a formulated diet three times daily at 08:00, 15:00, and 21:00. Activity and mortality were monitored daily; no significant mortality or abnormal stress behaviors were observed, confirming that the shrimp had fully adapted to the experimental conditions and that initial osmotic stress was eliminated.

Following acclimation, healthy individuals at the intermolt stage, with intact body structures and high vitality, were selected and stocked into both the pond and RAS systems at a uniform stocking density of 300 individuals per m^3^. A uniform feeding and management protocol was implemented for both groups throughout the entire culture cycle (no additional animal health products or water quality improvement products were used). The daily feeding rate was 4% of the estimated total shrimp biomass and the same feeding regime was strictly applied to both groups. Uneaten feed residues were siphoned out 1 h after each feeding to minimize additional water quality disturbance.

### 2.4. Environmental Monitoring and Coefficient of Variation (CV) Analysis

The late culture stage represents a critical phase in which shrimp biomass and system environmental load reach their peaks simultaneously, and cumulative environmental stress exerts the most pronounced effects on host physiology. Consequently, days 88 to 118 of the culture cycle were selected as the core observation window for water quality monitoring across all culture units.

Water samples (*n* = 5) were collected daily at 09:00 from a depth of 50 cm below the water surface. Seven key physicochemical indicators were measured using a multi-parameter water quality monitor (OCT-V, Octadem, Wuxi, China): total nitrogen, nitrite nitrogen, nitrate nitrogen, hydrogen sulfide, dissolved oxygen, temperature, and pH.

To scientifically quantify the degree of environmental fluctuation rather than relying solely on comparisons of mean values, coefficient of variation (CV) analysis was adopted. The CV value for each parameter was calculated with the formula CV = (σ/μ) × 100% (σ: the standard deviation, μ: the average value) [17,18]. A lower CV value indicates smaller parameter dispersion and reflects a higher level of environmental homeostasis during the late culture stage.

### 2.5. Calculation of Growth Performance Parameters

Based on the following formula, the growth performance indicators were calculated, including weight gain (WG), specific growth rate (SGR), feed conversion ratio (FCR), and survival rate (SR).

Weight gain (WG, g): $WG=W_{f}-W_{i}$

$W_{f}$ = final average body weight; $W_{i}$ = initial average body weight

Specific growth rate (SGR, %/d): $SGR=\frac{\ln W_{f}-\ln W_{i}}{t}\times 100\%$

t = total duration of the formal culture experiment

Feed conversion ratio (FCR): $FCR=\frac{F_{total}}{W_{gain_{total}}}$

$F_{total}$ = total actual feed intake; $W_{gain_{total}}$ = total weight gain of shrimp in each culture unit.

Survival rate (SR, %): $SR=\frac{N_{f}}{N_{i}}\times 100\%$

$N_{f}$ = final number of surviving shrimp; $N_{i}$ = initial stocking number

### 2.6. Sampling and Tissue Processing

All sampling was performed on day 118 at the end of the culture experiment, covering both environmental monitoring and biological detection samples. Operations followed strict aseptic protocols throughout the process to prevent sample contamination.

#### 2.6.1. Environmental Sampling

Environmental samples (*n* = 5) for TCBS-culturable *Vibrio*-like bacteria quantification were collected to evaluate the pathogen pressure of the two culture systems.

For water samples, a five-point composite sampling method was applied to minimize site-specific bias and ensure representativeness. Water was collected at 50 cm below the surface from the four corners and the center of each culture unit, then mixed in equal volumes to form a 500 mL composite sample per unit.

For solid-phase attachment matrices, sampling was tailored to the structural characteristics of each system. For the Pond group, composite surface sediment (top 5 cm) was collected using a sterile mud sampler. For the RAS group, which has no sediment in polypropylene culture tanks, mature biofilter media from the corresponding independent biochemical compartments were collected as the solid-phase matrix. Samples were randomly taken from five sites across different filter layers and thoroughly mixed for each replicate.

#### 2.6.2. Biological Sampling

Prior to sampling, all shrimp were fasted for 24 h to evacuate intestinal contents and avoid interference from residual feed in tissue assays.

Fifteen healthy individuals were randomly selected from the five culture units of each group for the measurement of total length (3 individuals per culture unit), body length, and body weight. From these, five typical healthy individuals at the intermolt stage were further selected (1 individuals per culture unit) for histological and biochemical analyses.

Hemolymph was collected from the ventral sinus using sterile syringes prefilled with EDTA anticoagulant (Bylabs, GL1813, Bylabs, Wuhan, China) and Plasma was obtained by centrifugation at 700× *g* for 10 min at 4 °C. Under aseptic conditions, gill, hepatopancreas, and muscle tissues were rapidly dissected. One portion of each tissue was immediately snap-frozen in liquid nitrogen and then stored at −80 °C for subsequent determination of tissue ammonia and immunobiochemical indicators. The remaining tissues were fixed in 4% paraformaldehyde (G1101, Servicebio, Wuhan, China) and 2.5% glutaraldehyde solution (G1102, Servicebio, Wuhan, China), respectively, for light microscopic and ultrastructural observations.

### 2.7. TCBS-Culturable Vibrio-like Bacteria Count

Total TCBS-culturable *Vibrio*-like bacteria in water and solid-phase matrices were quantified via the spread-plate method using commercial thiosulfate citrate bile salt sucrose (TCBS) agar plates (HBPM027, Hopebio, Qingdao, China). The assay was performed to compare environmental pathogenic bacterial pressure between the two culture modes, with five biological replicates per treatment (*n* = 5) and three technical replicates per sample.

For water samples, ten-fold serial dilutions were prepared directly with sterile physiological saline. A volume of 50 μL from the appropriate dilution was spread onto each agar plate and incubated at 28 °C for 24 h. Plates containing 30–300 colonies were selected for final counting. Bacterial counts were converted to colony-forming units per milliliter (CFU mL^−1^) using the formula:

Bacterial count (CFU mL^−1^) = Colony number × dilution factor × 20

For solid-phase matrices (pond surface sediment for the Pond group, mature biofilter media for the RAS group), 1.0 g of wet-weight sample was added to 9 mL of sterile saline (G3069, Servicebio, Wuhan, China) and thoroughly vortexed for 10 min to release attached bacteria into the liquid phase. The resulting supernatant was subjected to ten-fold serial dilution, and 50 μL of each dilution was spread onto TCBS plates following the same incubation and counting protocol. Bacterial counts were converted to colony-forming units per gram of wet sample (CFU g^−1^ wet weight) using the formula:

Bacterial count (CFU g^−1^) = Colony number × dilution factor × 200

### 2.8. Determination of Muscle Proximate Composition

The proximate composition of muscle samples (*n* = 5) was determined following standard Chinese national food safety analytical procedures. Moisture content was measured using the atmospheric pressure drying method at 105 °C. Crude protein content was determined using the Kjeldahl method. Crude lipid content was quantified by Soxhlet solvent extraction. Crude ash content was measured by gravimetric analysis after incineration in a muffle furnace at 550 °C.

### 2.9. Histopathological Observation and Morphometric Analysis

Tissue samples (*n* = 5) fixed in 4% paraformaldehyde were dehydrated through a graded ethanol series (C06915101, Naning reagent, Nanjing, China), cleared in xylene (C04305302, Naning reagent, Nanjing, China), and embedded in paraffin (C06915101, Naning reagent, Nanjing, China). Serial sections were cut at a thickness of 5 μm.

Hematoxylin and eosin (H&E) staining was performed according to the method described by Feldman et al. [19] to visualize general tissue architecture and histopathological alterations. Alcian blue-periodic acid-Schiff (AB-PAS) staining was conducted following the procedure of Kiliçarslan et al. [20] to detect glycogen and carbohydrate-rich components in hepatopancreatic tissues. Images were captured using a light microscope (Leica, Wetzlar, Germany). Digital morphometric analysis was performed using ImageJ software (v1.53, NIH, New York, NY, USA), with three representative fields randomly selected from each sample for quantification.

To evaluate the degree of hepatopancreatic tubule atrophy, the lumen area ratio (LAR) was calculated by delineating the total area of individual hepatopancreatic tubules (At) and their corresponding luminal area (Al), using the formula LAR (%) = (Al/At) × 100% [21,22]. For each biological replicate, 20 randomly selected intact hepatopancreatic tubules from three representative visual fields were measured, and the average LAR value was calculated to represent the degree of tubular atrophy for each sample.

For glycogen density quantification, magenta-positive signals from AB-PAS staining were isolated using the Color Deconvolution plugin. The mean optical density (MOD) of the target regions was then calculated to reflect the glycogen reserve status of the hepatopancreas [23,24].

### 2.10. Ultrastructural Observation

Ultrastructural analysis was performed on glutaraldehyde-fixed tissue samples to characterize subcellular morphological changes across the two culture modes.

#### 2.10.1. Scanning Electron Microscopy (SEM) Observation

Gill filaments and muscle samples (*n* = 5) were processed for SEM following the protocol described by Barbieri et al. [25]. Briefly, samples were rinsed with phosphate buffer (G0002, Servicebio, Wuhan, China) followed by dehydration through an ethanol gradient and drying at the critical point. After sputter coating with gold, specimens were examined under a SEM (SU8010, Hitachi, Tokyo, Japan).

#### 2.10.2. Transmission Electron Microscopy (TEM) Observation

Hepatopancreas tissues (*n* = 5) were prepared for TEM observation according to the method established by Cunningham et al. [26] method. Fixed tissues were post-fixed in osmium tetroxide (Sigma-Aldrich, Darmstadt, Germany), dehydrated in an ethanol gradient, and embedded in epoxy resin. Ultrathin sections were cut and double-stained with uranyl acetate and lead citrate, then viewed under a TEM (HT7800, Hitachi, Tokyo, Japan).

### 2.11. Detection of Tissue Ammonia Content and Biochemical Indicators

Tissue samples were homogenized in ice-cold sterile physiological saline (0.85%) at a ratio of 1:9 (*w*/*v*) to obtain 10% (*w*/*v*) homogenates. After centrifugation at 3000× *g* for 10 min at 4 °C, the supernatants were collected for subsequent analyses. All assays were performed using commercial kits from Nanjing Jiancheng Bioengineering Institute (Nanjing, China) according to the manufacturer’s instructions. Total soluble protein concentration in the supernatant was measured by the Coomassie brilliant blue method (A045-2-2) with bovine serum albumin (BSA) as the calibration standard. The following biochemical parameters were then determined: superoxide dismutase (SOD, A001-3-2), catalase (CAT, A007-1-1), malondialdehyde (MDA, A003-1-2), total antioxidant capacity (T-AOC, A015-2-1), lysozyme (LZM, A050-1-1), and tissue ammonia content (A086-1-1). Each assay included 5 biological replicates (*n* = 5) with 3 technical replicates per sample.

### 2.12. Statistical Analysis

All data are presented as mean ± standard deviation. Statistical analysis was performed using SPSSPRO (https://www.spsspro.com/). The Kolmogorov–Smirnov test and Levene’s test were first conducted to verify the normal distribution of data and homogeneity of variance between groups. When data satisfied the assumptions for parametric testing, independent samples *t* tests were applied to identify significant differences between the Pond and RAS groups. Significance levels were defined as follows: * *p* < 0.05, ** *p* < 0.01, and *** *p* < 0.001. Figures were generated using GraphPad Prism (version 9.0, San Diego, CA, USA) and Adobe Illustrator CC 2023 (San Jose, CA, USA).

## 3. Results

### 3.1. Dynamic Water Quality Characteristics and Quantitative Comparison of Fluctuation Degree Between Systems

**Figure 2:**
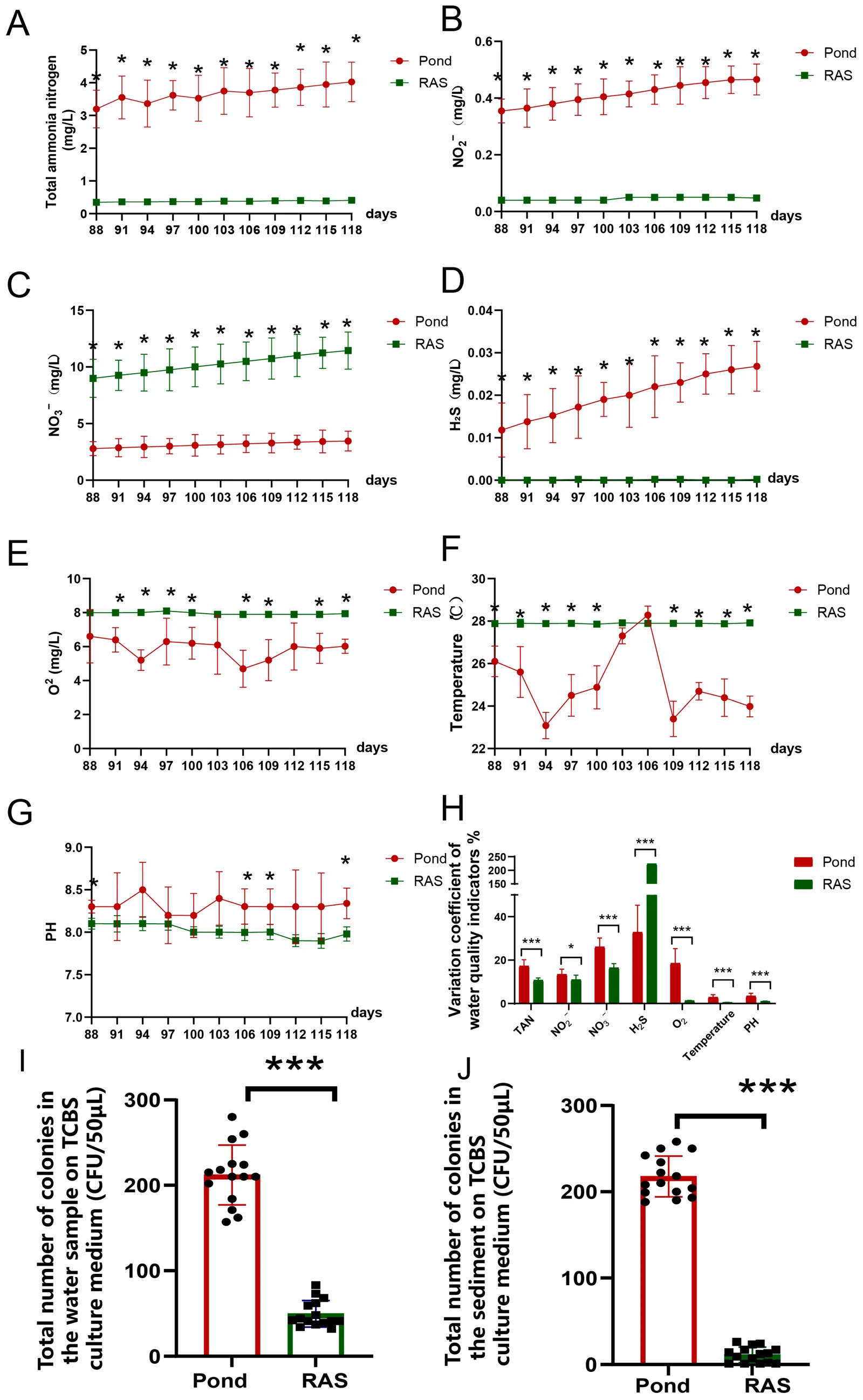
Water quality dynamics, environmental homeostasis and TCBS-culturable *Vibrio*-like bacterial counts between the Pond and RAS groups. (**A**–**G**)Trends of key physical and biochemical indicators over time including TAN (**A**), nitrite nitrogen (**B**), nitrate nitrogen (**C**), hydrogen sulfide (**D**), dissolved oxygen (**E**), temperature (**F**), and pH (**G**). (**H**) Comparison of CV values for major water quality indicators between the Pond and RAS groups. (**I**) Comparison of TCBS-culturable *Vibrio*-like bacteria counts in water samples (CFU mL^−1^). (**J**) Comparison of TCBS-culturable Vibrio-like bacteria counts in solid-phase matrices (CFU g^−1^ wet weight; pond sediment for the Pond group, biofilter media for the RAS group). * *p* < 0.05, ** *p* < 0.01, and *** *p* < 0.001.

During continuous monitoring from day 88 to day 118, the RAS group exhibited markedly superior water quality stability compared with the Pond group. In the RAS, TAN (Figure 2A) and nitrite nitrogen (Figure 2B) remained consistently low, while nitrate nitrogen increased gradually in a stepwise manner (Figure 2C), indicating that the biofilter maintained sustained nitrification activity throughout the late culture period. Additionally, the hydrogen sulfide (H_2_S) concentration in the RAS remained near the detection limit (Figure 2D). In the Pond group, by contrast, TAN and nitrite nitrogen accumulated progressively over time, accompanied by pronounced daily fluctuations in dissolved oxygen (Figure 2E), temperature (Figure 2F), and pH (Figure 2G).

To quantify the degree of environmental fluctuation, we calculated the coefficient of variation (CV) for each physicochemical indicator. The CV values for nearly all parameters in the RAS group were substantially lower than those in the Pond group (Figure 2H), confirming that the RAS environment experienced considerably less physicochemical disturbance. H_2_S was the only parameter for which the CV appeared elevated; this was an artifact of concentrations nearing the detection limit, rather than an indication of genuine instability.

Regarding microbial pressure, TCBS-culturable *Vibrio*-like bacteria loads in the water (Figure 2I) and solid phase attachment matrices (Figure 2J) of the Pond group were higher than those of the RAS group. This elevated pathogen load, combined with the physicochemical instability described above, posed a considerable biosecurity threat to shrimp reared in the open-pond system.

### 3.2. Analysis of Clinical Gross Traits and Growth Performance

**Figure 3:**
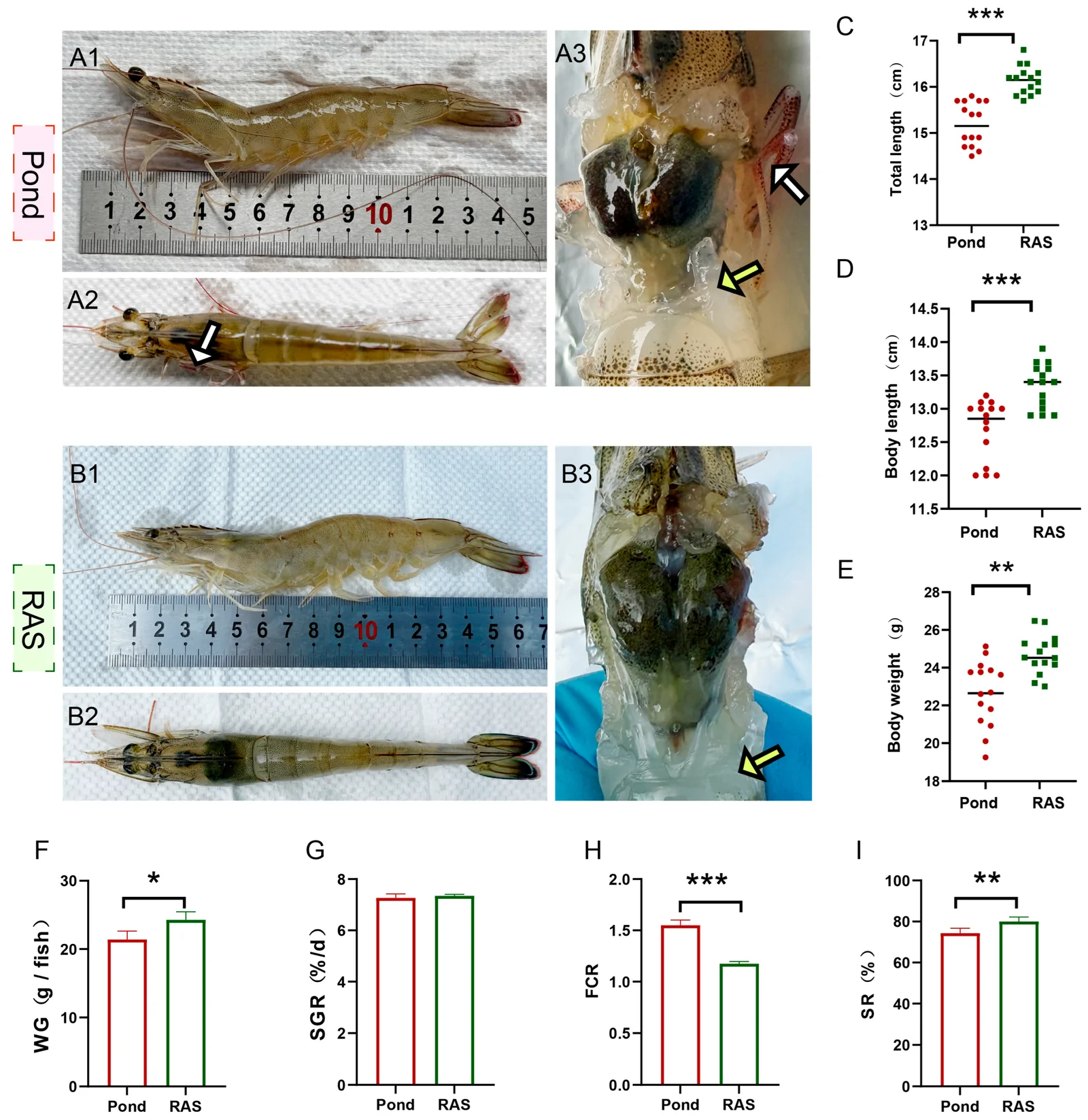
Comparison of gross morphological characteristics and growth performance between the Pond and RAS groups. (**A1**–**A3**) Gross morphology of the Pond group, showing brownish yellow body color and abnormal redness of appendages (white arrows) along with characteristic muscle transparency (yellow arrows). (**B1**–**B3**) Gross morphology of the RAS group, showing healthy dark brown hepatopancreas and firm, plump muscle texture (yellow arrows). (**C**–**E**) Statistical distribution of growth indicators including total length (**C**), body length (**D**), and body weight (**E**). (**F**–**I**) Statistical distribution of growth performance parameters including Weight gain (WG) (**F**), specific growth rate (SGR) (**G**), feed conversion ratio (FCR) (**H**), and survival rate (SR) (**I**). * *p* < 0.05, ** *p* < 0.01, and *** *p* < 0.001.

At the end of the culture experiment (day 118), the two rearing modes produced marked differences in the external phenotype and growth performance of *L. vannamei*. Shrimp in the Pond group displayed obvious subhealth signs, including a brownish yellow body coloration and abnormal reddish discoloration of the periopods and pleopods (Figure 3(A1–A3)). These clinical signs suggested environmental stress or potential *Vibrio* infection. Anatomical examination further revealed that the hepatopancreas of pond-reared shrimp was visibly atrophied and loose in texture, while the abdominal muscle exhibited a characteristic translucent appearance (Figure 3(A3)). In contrast, shrimp reared in RAS maintained a normal body color, a firm and plump hepatopancreas with a healthy dark brown shade, and compact muscle texture (Figure 3(B1–B3)).

Growth performance data corroborated these visual observations. The RAS group showed significant advantages over the Pond group in all three measured parameters: total length (Figure 3C), body length (Figure 3D), and body weight (Figure 3E) were all substantially higher in the RAS group, indicating that the more controlled rearing environment promoted superior somatic growth.

In addition, shrimp reared in the RAS system exhibited significantly higher WG (Figure 3F) and SR (Figure 3I) than those in the open-pond system. The FCR (Figure 3H) was significantly lower in the RAS group compared with the Pond group. A trend toward higher SGR (Figure 3G) was also observed in the RAS group, but no statistically significant difference was detected between the two groups.

### 3.3. Histopathological and Ultrapathological Analysis of Gills

**Figure 4:**
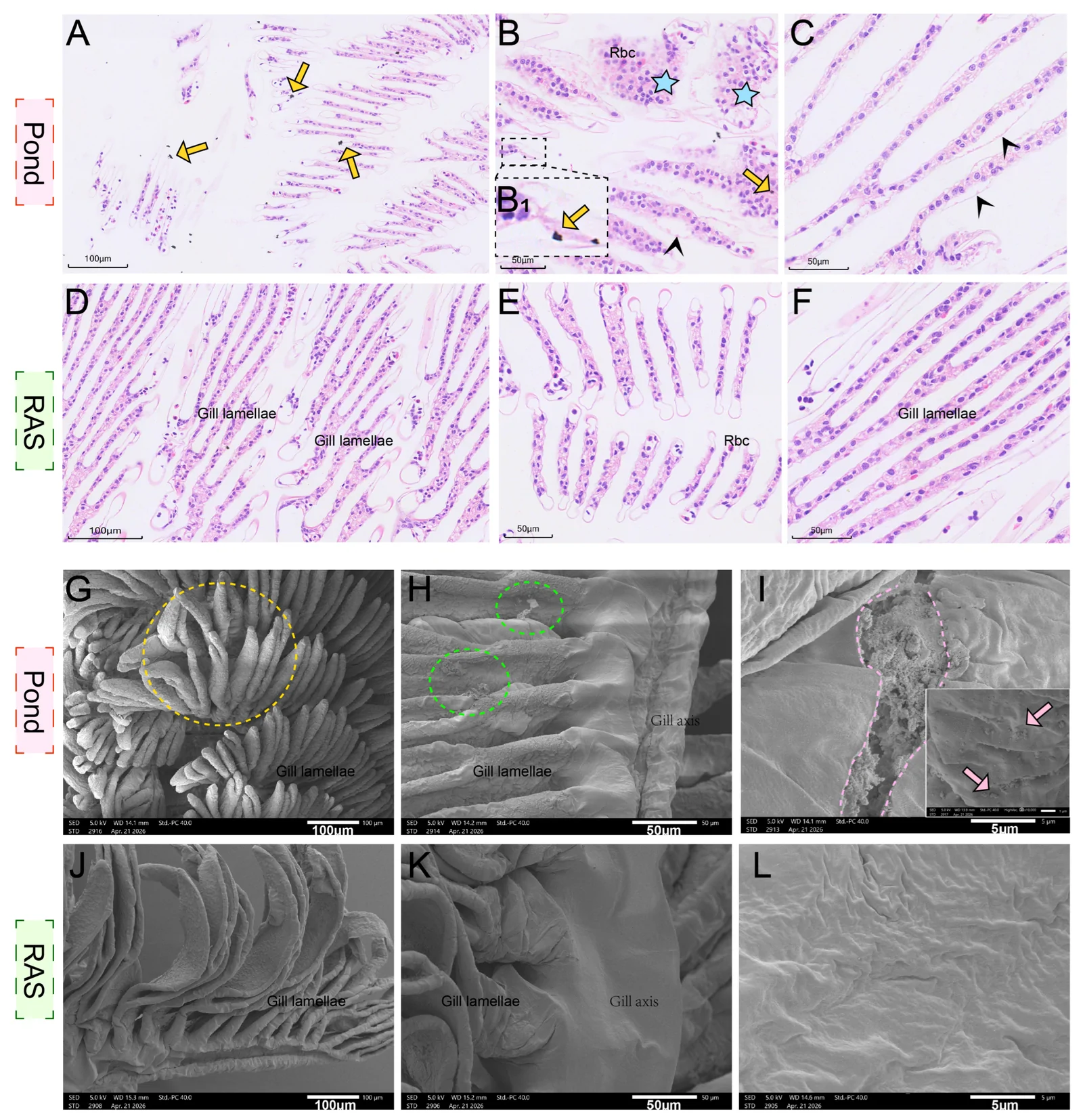
Comparison of light microscopic structure and SEM ultrastructural characteristics of gill tissues between the Pond and RAS groups. (**A**–**C**) H&E stained sections of the Pond group. (**A**) Gill tissue showing environmental debris and organic particles in the interlamellar spaces (yellow arrows). (**B**) Interlamellar debris, gill lamellae fusion (blue stars) and epithelial detachment (black arrowheads). (**B1**) Higher magnification view of the boxed area in panel (**B**). (**C**) Epithelial detachment of gill lamellae (black arrowheads). (**D**–**F**) H&E stained sections of the RAS group. (**D**,**E**) Regular arrangement of gill lamellae. (**F**) Intact morphology of gill lamellae. (**G**–**I**) SEM observations of the Pond group. (**G**) Gill lamellar hyperplasia and interstitial adhesion (yellow dashed circle). (**H**) Epithelial shedding on gill surface (green dashed circles). (**I**) Epithelial fracture (pink dashed circle) and bacterial attachment (pink arrows; inset shows magnified view of bacteria). (**J**–**L**) SEM observations of the RAS group. (**J**,**K**) Orderly arranged gill lamellae attached to the gill axis. (**L**) Smooth epithelial surface of gill lamellae. Abbreviation: Rbc, red blood cells. Scale bars are marked on each panel.

Histological examination revealed marked differences in gill structure between the two rearing groups. In the Pond group, gill filaments exhibited severe pathological alterations (Figure 4A–C). The interlamellar spaces were extensively occupied by dark particulate matter, including organic debris and algal particles, which formed a physical barrier obstructing normal water flow across the respiratory surface (Figure 4A,B). In some regions, adjacent gill lamellae had undergone fusion, accompanied by widespread detachment and sloughing of the respiratory epithelium (Figure 4C). By contrast, gill filaments in the RAS group were arranged in a dense, orderly fashion, with clean surfaces and well-preserved respiratory epithelial integrity (Figure 4D–F).

SEM observations provided additional ultrastructural detail. In the Pond group, hyperplastic changes in the gill lamellae led to interstitial adhesion, creating a physical obstruction to waste excretion (Figure 4G). Extensive epithelial cell detachment and fracture were evident (Figure 4H), and prominent bacterial colonies were observed adhering to the lamellar surfaces (Figure 4I). In sharp contrast, the gill lamellae of RAS-reared shrimp appeared plump, upright, and uniformly arranged along the gill axis, with smooth surfaces free from visible pathogen colonization or debris attachment (Figure 4J–L).

### 3.4. Histopathological and Metabolic Ultrastructural Characteristics of the Hepatopancreas

**Figure 5:**
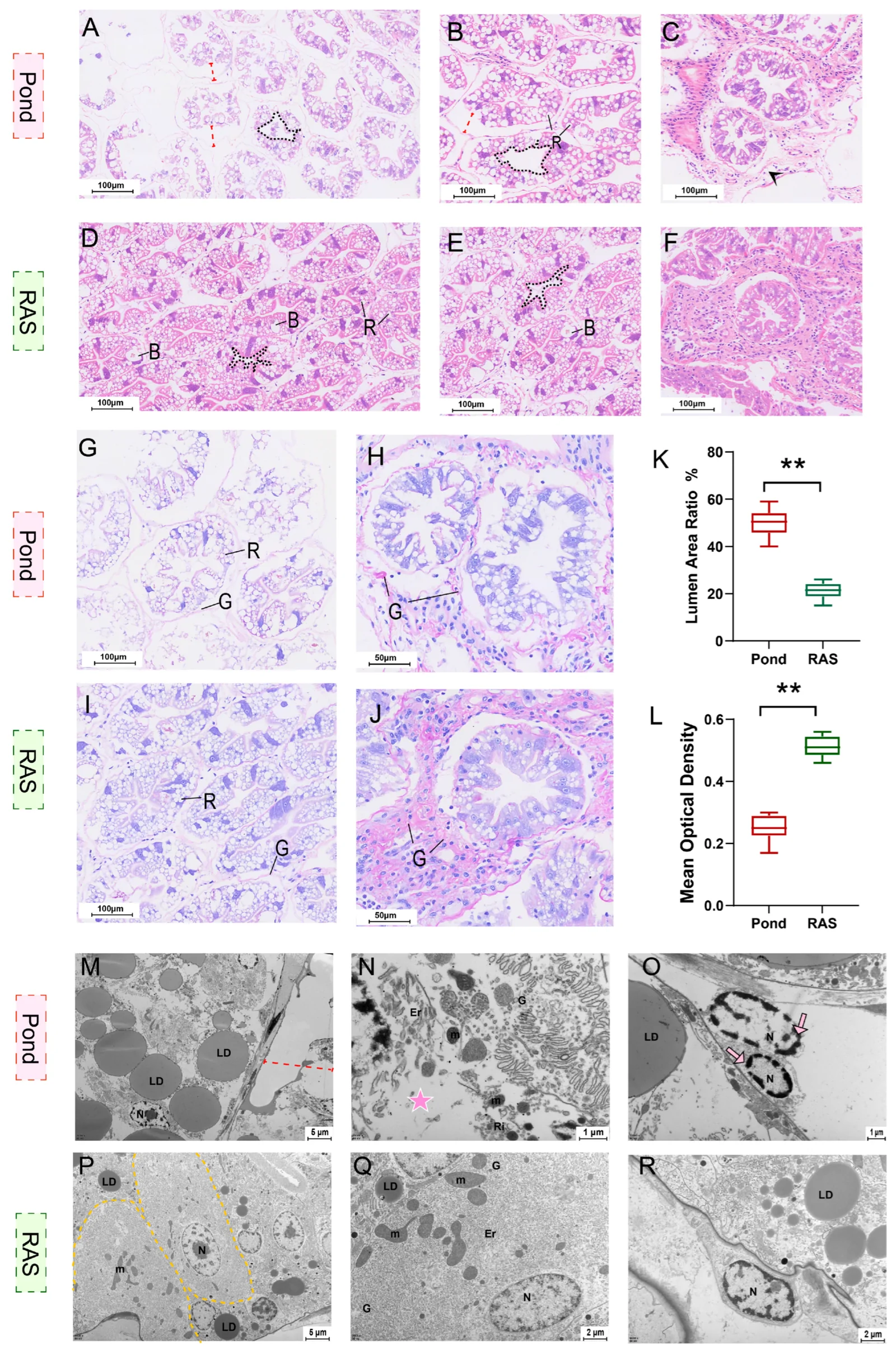
Comparison of histological H&E and AB-PAS characteristics of hepatopancreas between the Pond and RAS groups. (**A**–**C**) H&E stained sections of the Pond group. (**A**,**B**) Showing detachment between the epithelial layer and basal membrane (red dashed arrows). and distorted, irregular tubule lumens (black dashed lines). (**C**) Showing rupture of intertubular connective tissue (black arrowheads). (**D**–**F**) H&E stained sections of the RAS group. (**D**,**E**) Showing dense and intact hepatopancreatic tissue and typical star-shaped tubule lumens (black dashed lines). (**F**) Showing compact and well-preserved tubular structure. (**G**,**H**) AB-PAS stained sections of the Pond group, showing significant glycogen depletion. (**I**,**J**) AB-PAS stained sections of the RAS group, showing abundant glycogen reserves. (**K**) Quantitative comparison of lumen area ratio (LAR). (**L**) Quantitative comparison of mean optical density (MOD) of magenta. (**M**–**O**) TEM images of the Pond group. (**M**) Low-magnification view of absorptive cells, showing abnormally fused oversized lipid droplets and dilated intercellular space (red dashed line). (**N**) High-magnification view of cytoplasmic organelles, showing disorganized endoplasmic reticulum and degraded cytoplasmic matrix (pink star). (**O**) Cell nucleus with marked heterochromatin margination and pyknosis (pink arrows). (**P**–**R**) TEM images of the RAS group. (**P**) Low-magnification view of intact absorptive cells (yellow dashed line), with evenly distributed small lipid droplets and abundant mitochondria. (**Q**) High-magnification view of cytoplasm, showing well-arranged endoplasmic reticulum, intact mitochondria and abundant glycogen granules. (**R**) Regularly shaped cell nucleus with uniformly distributed chromatin. * *p* < 0.05, ** *p* < 0.01, and *** *p* < 0.001. R, absorptive cells (R cells) rich in lipid droplets; B, secretory cells (B cells) with large secretory vacuoles; G, glycogen; LD, lipid droplet; N, nucleus; Er, endoplasmic reticulum; m, mitochondrion; Ri, ribosome.

Histological evaluation of the hepatopancreas further confirmed the deleterious effects of chronic environmental fluctuation on this metabolic center. H&E staining revealed prominent histopathological degeneration in the Pond group. Hepatopancreatic tubules underwent widespread atrophy, with clear detachment of the epithelial layer from the underlying basement membrane (Figure 5A,B). The intertubular connective tissue lost structural continuity and appeared disorganized, and tubule lumens were distorted and irregularly dilated (Figure 5C). Concurrently, the abundance of secretory B-cells and storage R-cells in the tubular epithelium was markedly decreased (Figure 5B), implying compromised digestive and storage functions. In sharp contrast, the RAS group maintained a compact and well-organized hepatopancreatic architecture, with typical star-shaped tubule lumens and tightly aligned epithelial cells (Figure 5D–F). The epithelial cytoplasm was filled with abundant lipid droplets and secretory vacuoles, indicative of active metabolic and secretory activity.

Glycogen reserves in hepatopancreatic tissues were visualized via AB-PAS histochemical staining, with results aligned with the histological findings. In the RAS group, hepatocytes exhibited intense, homogeneous magenta-positive signals, demonstrating abundant glycogen deposition (Figure 5I,J). In the Pond group, however, glycogen was severely depleted, with only sparse, fragmented positive signals detectable in the atrophic epithelial layer (Figure 5G,H).

Quantitative morphometric analysis corroborated these qualitative observations. The lumen area ratio (LAR), a quantitative indicator of tubular atrophy, was significantly higher in the Pond group than in the RAS group (Figure 5K), confirming severe tubule shrinkage under fluctuating conditions. Correspondingly, the mean optical density (MOD) of AB-PAS positive signals, a direct proxy for glycogen content, was significantly lower in the Pond group (Figure 5L), reflecting a dramatic reduction in energy reserves.

TEM observations further revealed subcellular structural differences in hepatopancreatic R cells between the two groups. In the Pond group, R-cells contained abnormally fused, oversized lipid droplets, with sparse cytoplasmic matrix and reduced functional organelles (Figure 5M). The intercellular space was markedly dilated. At higher magnification, the endoplasmic reticulum was disorganized and vesicularly dilated, mitochondrial cristae were blurred, and the cytoplasmic matrix showed degraded vacuolation (Figure 5N). Cell nuclei presented typical pyknosis, with massive heterochromatin marginally distributed along the nuclear membrane (Figure 5O).

In the RAS group, R-cells had plump morphology with evenly dispersed small lipid droplets and dense cytoplasmic matrix (Figure 5P). High-magnification observation showed neatly stacked endoplasmic reticulum, mitochondria with intact and clear cristae, and abundant glycogen granules scattered in the cytoplasm (Figure 5Q). Cell nuclei were regular in shape, with uniformly distributed chromatin and no signs of pyknosis (Figure 5R).

### 3.5. Comparison of Muscle Tissue Structure and Nutritional Composition

**Figure 6:**
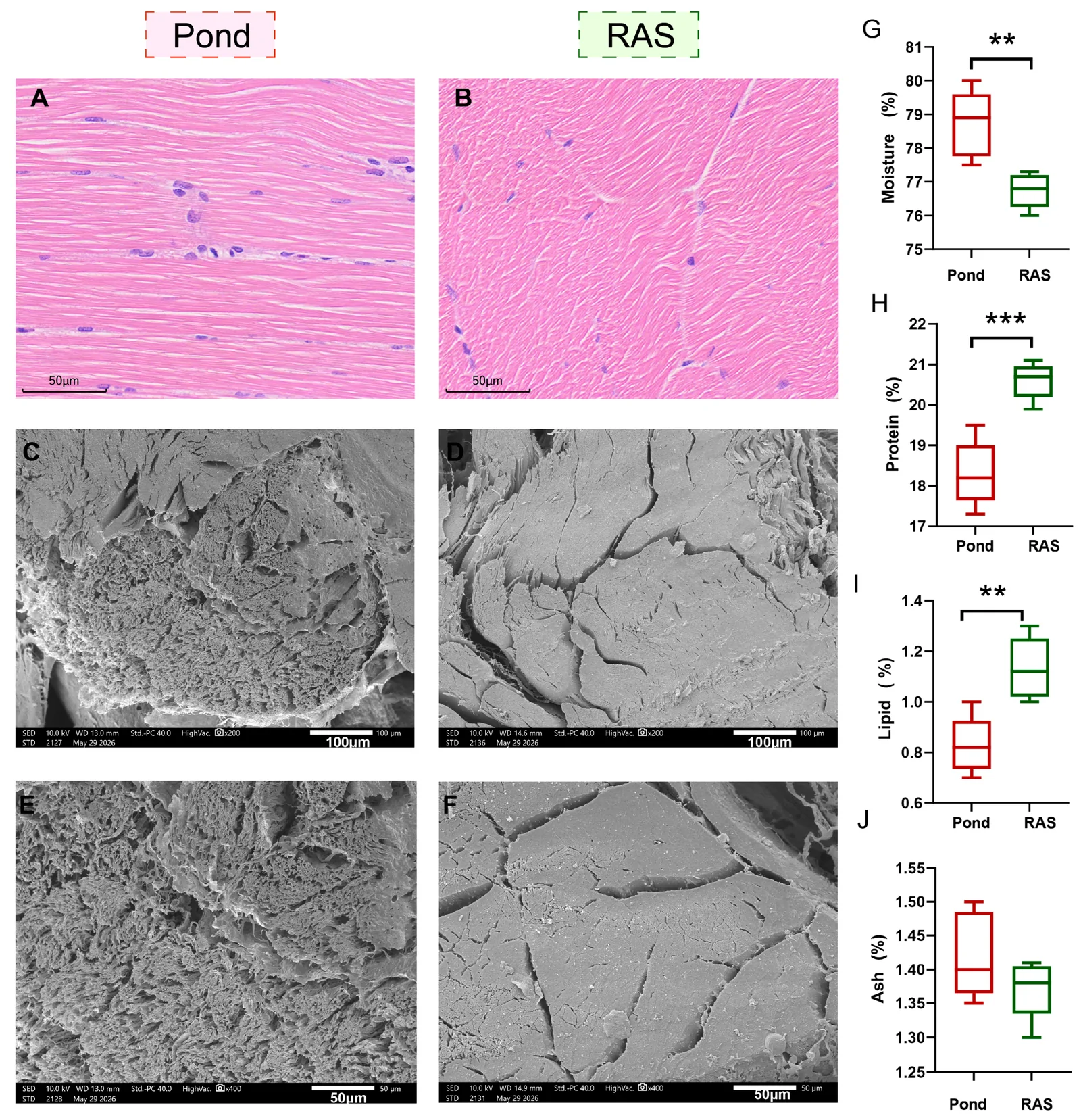
Comparison of muscle tissue structure and nutritional composition between the Pond and RAS groups. (**A**,**B**) H&E stained sections of striated muscle in the Pond group (**A**) and the RAS group (**B**). (**C**–**F**) SEM observations of muscle cross-sections. Low magnification views (**C**,**D**) show typical porous and loose features in the Pond group (**C**) and a dense morphology in the RAS group (**D**). High magnification views (**E**,**F**) reveal prominent structural gaps between myofibril bundles in the Pond group (**E**) compared to the highly compact and continuous fibers in the RAS group (**F**). (**G**–**J**) Nutritional composition of muscle including moisture (**G**), crude protein (**H**), crude lipid (**I**), and crude ash (**J**). * *p* < 0.05, ** *p* < 0.01, and *** *p* < 0.001.

The two rearing modes also exerted distinct effects on muscle tissue architecture and quality characteristics. Histological examination of striated muscle revealed that muscle fibers in the Pond group were loosely arranged, with noticeable interbundle spaces and a general loss of compactness (Figure 6A). In the RAS group, by contrast, muscle fiber bundles were arranged tightly, and in a highly ordered manner with clear and regular striated structures, demonstrating excellent tissue density (Figure 6B).

SEM of muscle cross-sections provided further ultrastructural evidence of quality degradation. At both low and high magnifications, the Pond group exhibited a characteristic porous and loose architecture (Figure 6C,E). Prominent structural gaps were observed between myofibril bundles, and the matrix surface appeared rough and discontinuous. In the RAS group, muscle cross-sections presented as an extremely compact and continuous whole, with virtually no visible intermyofibrillar spaces (Figure 6D,F).

Biochemical composition analysis corroborated these structural observations. Muscle from the RAS group displayed a nutrient deposition profile characterized by lower moisture (Figure 6G), higher crude protein (Figure 6H), and higher crude lipid content (Figure 6I) compared with the Pond group. Crude ash content, however, showed no statistically significant difference between the two groups (Figure 6J).

### 3.6. Analysis of Tissue Ammonia Accumulation, Oxidative Stress, and Nonspecific Immune Levels

**Figure 7:**
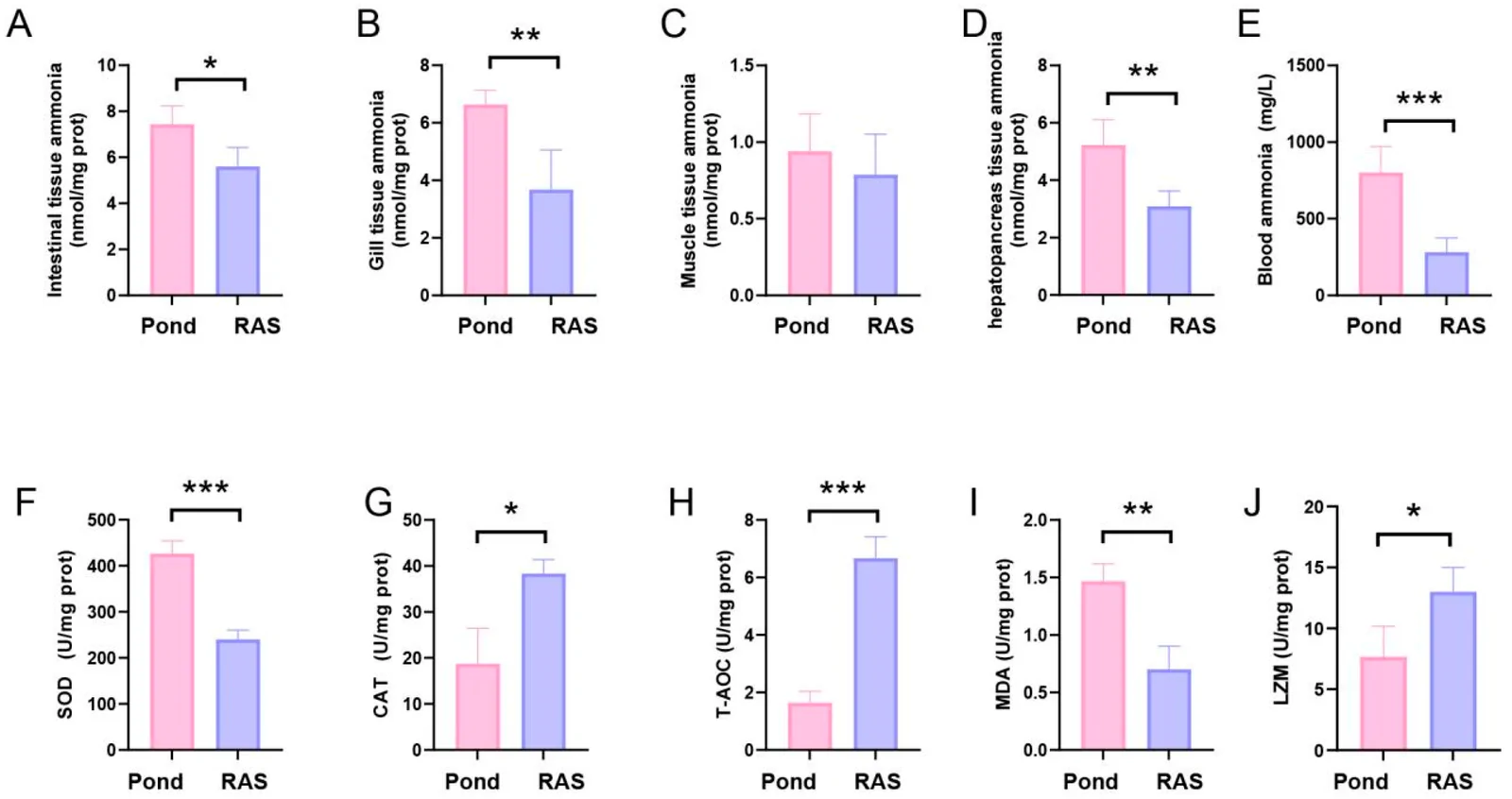
Comparison of tissue ammonia accumulation, oxidative stress indicators, and nonspecific immune enzyme activities between the Pond and RAS groups. (**A**–**E**) Ammonia levels in different tissues and hemolymph including intestine (**A**), gills (**B**), muscle (**C**), hepatopancreas (**D**), and hemolymph ammonia (**E**). (**F**–**I**) Indicators of oxidative stress and antioxidant capacity including SOD (**F**), CAT (**G**), T-AOC (**H**), and MDA (**I**). (**J**) Comparison of lysozyme LZM activity between the two groups. * *p* < 0.05, ** *p* < 0.01, and *** *p* < 0.001.

Biochemical assays confirmed that shrimp in the Pond group, subjected to prolonged environmental instability, had developed systemic tissue ammonia accumulation (Figure 7A–E). Ammonia concentrations in the hemolymph (Figure 7E), intestine (Figure 7A), gills (Figure 7B), and hepatopancreas (Figure 7D) were all significantly higher in the Pond group than in the RAS group. Muscle tissue ammonia, however, showed no significant difference between the two groups (Figure 7C).

Plasma oxidative stress markers also differed between the two groups. Regarding redox homeostasis indicators, SOD activity (Figure 7F) and MDA content (Figure 7I) were significantly higher in the Pond group than in the RAS group, whereas CAT activity (Figure 7G) and total antioxidant capacity (T-AOC, Figure 7H) were significantly lower. Plasma lysozyme (LZM) activity, a key indicator of nonspecific immune competence, was significantly lower in the Pond group than in the RAS group (Figure 7J), suggesting a suppressed immune status in pond-reared shrimp under the tested conditions.

## 4. Discussion

To place the findings of this study in the context of existing research, we compared key experimental conditions and growth performance outcomes with representative studies on *L. vannamei* cultured in different systems (Table 1). Overall, the growth advantage of more controlled, closed culture systems (RAS) relative to open earthen ponds observed in this study is broadly consistent with the consensus established by previous studies across different geographic regions and salinity conditions. However, most existing comparative studies were conducted under mismatched geographic, climatic, or hydrological backgrounds between culture systems, which may introduce confounding factors unrelated to the culture mode itself. A key distinction of this study is the in situ RAS modification design, which minimizes macro-environmental confounding and enables a more reliable comparison of physiological responses. Furthermore, we extend beyond production metrics to multi-scale histological and physiological mechanisms underlying the performance differences.

### 4.1. Water Quality Characteristics and Microbial Pressure of the Two Culture Modes

Water quality stability is a primary extrinsic factor determining the physiological condition of cultured shrimp. The pronounced water quality instability in open ponds stems from their reliance on natural ecological self-purification, which offers limited buffering capacity against weather shifts and feed loading.

Such fluctuations act as a sustained physical stressor, forcing shrimp to continuously expend metabolic energy on internal homeostasis. In addition to physicochemical instability, the Pond group also carried significantly higher TCBS-culturable *Vibrio*-like bacteria count in both water and sediment, representing a substantial biosecurity risk. These elevated pathogen levels were consistent with the clinical signs observed in pond-reared shrimp, such as reddish appendages. Taken together, the combination of high environmental variability and high pathogen pressure appears to constitute the primary bottleneck of the open-pond model.

### 4.2. Structural Damage and Functional Impairment of the Gill Tissue Barrier

Gills serve as the primary interface between shrimp and their aquatic environment, and their structural integrity is essential for respiration, osmoregulation, and waste excretion [27,28,29]. In the Pond group, the interlamellar spaces were extensively clogged with organic debris and algal particles.

This physical obstruction not only blocks water flow across the respiratory surface, but also directly occludes the outward excretion route for endogenous ammonia, which is mainly eliminated through the gill epithelium [30]. Concurrently, the pond water contained persistently high ambient ammonia concentrations, and the damaged gill barrier allowed external ammonia to diffuse inward more readily [31,32]. The convergence of blocked efflux and enhanced influx thus provides a coherent explanation for the systemic tissue ammonia accumulation observed in pond-reared shrimp, particularly in the hemolymph, intestine, and hepatopancreas. The absence of ammonia elevation in muscle tissue, by contrast, suggests that ammonia distribution among tissues may not be uniform, with metabolically active organs appearing more susceptible to accumulation.

Beyond physical clogging, SEM observations revealed two additional signs of gill compromise in the Pond group: bacterial colonization on the lamellar surface, and extensive detachment of the respiratory epithelium. Both findings point to a breakdown of the epithelial barrier. When the epithelium is lost or disrupted, the underlying basement membrane and connective tissue become exposed, creating potential entry routes for opportunistic pathogens [13]. The concurrent presence of bacterial colonies on the damaged surface suggests that local defense mechanisms (such as mucus secretion) may have been insufficient to prevent microbial adherence. In the RAS group, by contrast, the gill surface remained free of adherent microbes and the epithelium was intact. This clean, well-organized structure supports normal respiratory and excretory functions and likely reduces the likelihood of pathogen entry through the gill route.

### 4.3. Hepatopancreas Metabolic Damage and Changes in Energy Reserves

The hepatopancreas is the primary site of metabolism, nutrient storage, and detoxification in shrimp [33,34], and its structural integrity is critical for maintaining physiological homeostasis. In the Pond group, hepatopancreatic tubules displayed pronounced atrophy, reflected in a significantly increased LAR, along with detachment of the epithelial layer from the basement membrane. These structural abnormalities were accompanied by a marked reduction in the numbers of secretory B-cells and storage R-cells.

B-cells are characterized by large secretory vacuoles and are involved in nutrient absorption and intracellular digestion, whereas R-cells contain a large number of lipid droplets and are responsible for lipoprotein synthesis, nutrient transport, and heavy-metal detoxification [33,35]. The depletion of these cell types in the Pond group thus points to a compromised capacity for both nutrient processing and reserve accumulation. The RAS group, by contrast, maintained compact tubule architecture with well-developed epithelial cells and abundant cytoplasmic inclusions.

The functional consequences of this structural deterioration were evident at both the histochemical and ultrastructural levels. AB-PAS staining revealed severe glycogen depletion in the hepatopancreas of pond-reared shrimp, whereas the RAS group maintained abundant glycogen reserves. Glycogen serves as the primary rapidly mobilizable energy reserve in crustacean hepatopancreas, providing an immediate source of glucose for ATP production under acute stress conditions [36]. In the Pond group, the persistent environmental fluctuations likely imposed repeated or sustained energy demands that continuously drew upon this reserve. The depletion of glycogen stores suggests that the frequency and intensity of these energy demands exceeded the capacity for repletion, leaving the hepatopancreas in a state of chronic energy deficit [37]. Compounding this issue, the loss of R cells and B cells would further impair the capacity for nutrient uptake and storage, reducing the availability of precursor materials for glycogen synthesis and replenishment.

Concurrently, TEM observations showed that mitochondria in the hepatopancreatic cells of the Pond group exhibited cristae shrinkage and breakage, matrix vacuolization, and disrupted membrane integrity. Mitochondrial damage would directly compromise oxidative phosphorylation and ATP synthesis [38], meaning that even when glycogen is mobilized and broken down, the downstream energetic yield remains limited. The concurrent depletion of the energy reserve (glycogen) and impairment of the energy production machinery (mitochondria) thus creates a double bottleneck in cellular energy supply. This combined deficiency provides a mechanistic explanation for the atrophy, structural loosening, and growth suppression observed in pond-reared shrimp. In the RAS group, by contrast, both glycogen stores and mitochondrial ultrastructure remained intact, indicating that the more controlled culture environment allowed the hepatopancreas to maintain full metabolic capacity without excessive diversion of energy toward stress responses and tissue repair.

### 4.4. Redox Imbalance and Suppressed Humoral Immunity Induced by Tissue Ammonia Accumulation

Excessive tissue ammonia accumulation is a well-established trigger of oxidative stress [39]. In the Pond group, the elevated ammonia levels in hemolymph and multiple tissues likely promoted overproduction of reactive oxygen species, leading to the observed redox imbalance [40]. The concurrent elevation of SOD activity and MDA content in plasma is particularly telling: SOD upregulation represents an initial compensatory response to superoxide anion overload, yet the simultaneous increase in MDA indicates that this response was insufficient to prevent lipid peroxidation damage [38]. This pattern of elevated SOD and MDA, paired with reduced CAT and total antioxidant capacity, indicates the antioxidant system is operating at near capacity and cannot fully neutralize the oxidative burden imposed by elevated tissue ammonia [41].

The reduced plasma lysozyme activity observed in the Pond group, occurring against a background of elevated pathogen load and severe oxidative stress, is indicative of impaired nonspecific humoral immune function. Similar findings have been reported in *L. vannamei* under chronic ammonia exposure, where immune parameters declined concurrently with oxidative stress markers [42]. One plausible pathway linking these observations is that oxidative damage to hemocytes compromises their phagocytic and bactericidal functions, ultimately reducing lysozyme secretion and undermining systemic immune surveillance [43]. In the RAS group, by contrast, the more controlled rearing conditions sustained lower ammonia levels and milder oxidative conditions, and plasma LZM activity remained significantly higher than that of the Pond group. This pattern effectively avoided the immunosuppression associated with redox imbalance, thereby safeguarding the basal immune competence of the organism.

### 4.5. Logical Correlation Between Growth Performance and Muscle Nutrient Deposition

Differences in rearing conditions between the two culture modes, mediated through the physiological mechanisms discussed above, ultimately result in divergent growth performance and muscle quality in *L. vannamei* [44].

In the Pond group, frequent physicochemical fluctuations and persistent tissue ammonia accumulation constitute sustained chronic stress. According to crustacean energy budget frameworks [45,46], such conditions shift the allocation of metabolic resources toward homeostatic maintenance, detoxification and tissue repair, leaving less energy available for somatic growth and protein synthesis. This pattern likely accounts for the reduced SGR, elevated FCR, lower final weight and decreased muscle protein content observed in pond-reared shrimp [47]. Ultrastructural observations further support this interpretation: the porous, loose myofibrillar architecture with enlarged inter-fibrillar spaces likely reflects impaired protein deposition, with void spaces occupied by water, which aligns with the higher moisture and lower crude protein measured in these samples [48].

In the RAS group, by contrast, lower overall stress load corresponds to more efficient growth and muscle deposition. It is plausible that reduced demand for stress defense and tissue repair allows ingested nutrients to be channeled more efficiently into muscle tissue components, resulting in denser myofibrils, lower moisture, and higher crude protein and lipid content.

**Figure 8:**
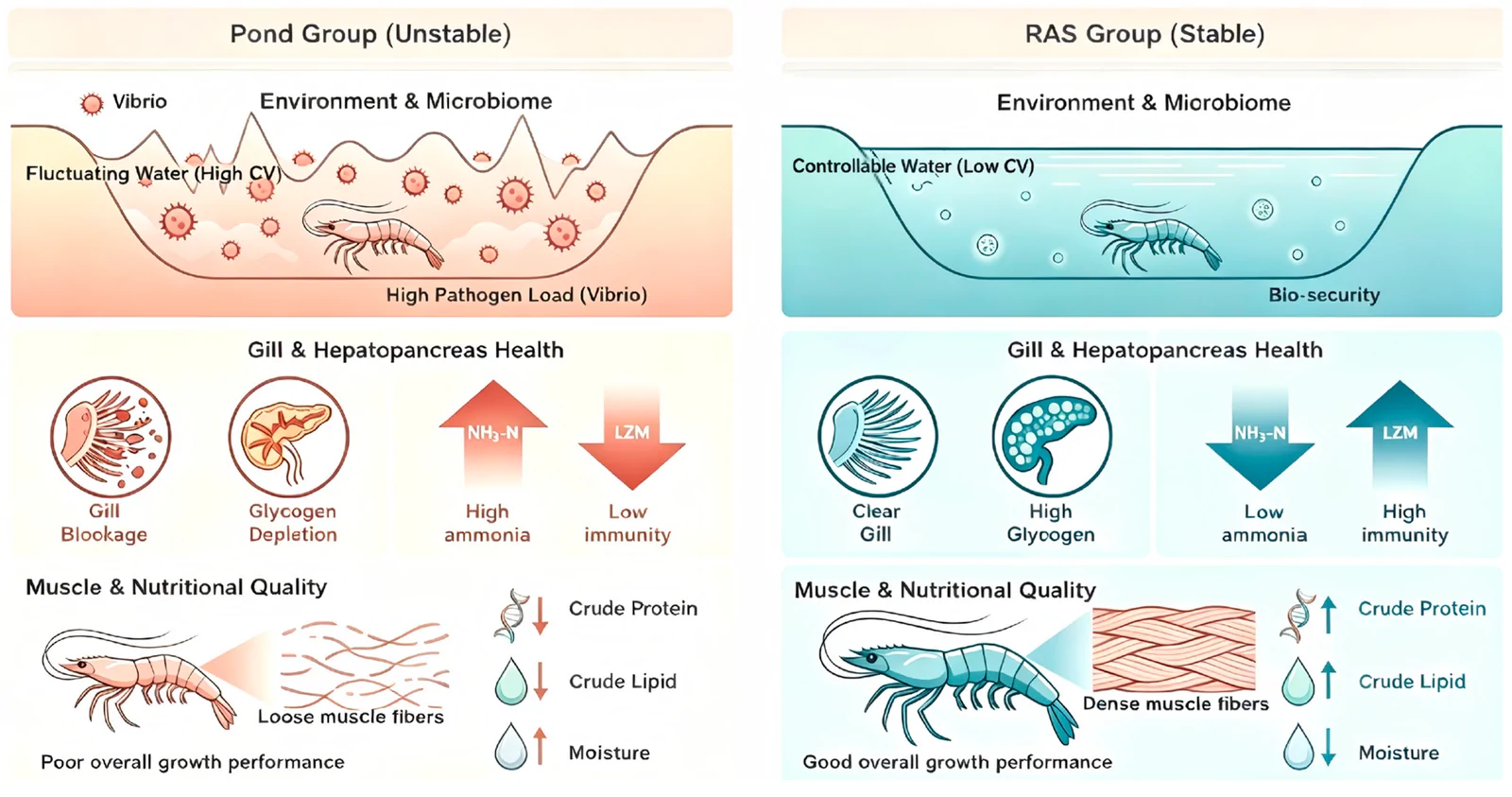
Comprehensive schematic diagram illustrating physiological homeostasis and muscle quality differences between the two culture modes. The schematic compares the cascade effects between the Pond model (left side) and the RAS model (right side) across environmental factors, organ integrity, physiological stress responses and final growth quality.

To integrate the above findings, we propose a schematic model illustrating how culture modes shape the physiological health and muscle quality of *L. vannamei* (Figure 8). The model illustrates that combined stressors in open ponds drive tissue damage and metabolic disruption that divert resources away from growth [49], whereas the lower stress burden in RAS preserves tissue function and supports efficient nutrient partitioning.

These performance and quality differences carry clear economic implications for commercial production. RAS can reduce operating costs through improved feed efficiency and shorter culture cycles, while higher product quality and lower disease risk may support premium pricing and reduce production volatility. However, higher upfront capital expenditure, continuous system energy consumption and demand for skilled management remain notable barriers to large-scale adoption. Traditional open ponds, by comparison, have lower initial investment and simpler operational requirements, making them more accessible for small-scale producers despite higher environmental risk and more variable product quality.

### 4.6. Study Limitations and Future Research Directions

While the experimental design has controlled for major manageable variables such as initial shrimp size, feed formulation, feeding regime, stocking density and culture duration to ensure the comparability between groups, there are still boundaries to the scope of inference and depth of mechanism analysis. These constraints also point to key avenues for future investigation.

First, this study compares two intact culture systems, where multiple environmental factors co-vary including fluctuation amplitude, pathogen load, water exchange and suspended solids. Therefore, the independent contribution of any single factor to the observed phenotypes cannot be quantitatively isolated from the current dataset. Disentangling the relative roles of these individual drivers will require targeted controlled experiments in future work.

Second, the immune assessment in this study focused on classical humoral biochemical markers represented by lysozyme activity, which provides a reliable indicator of overall nonspecific immune status. However, cellular immune parameters and the molecular regulatory pathways underlying the observed responses remain unexplored.

Third, our interpretation of differential metabolic energy allocation is built on coherent phenotypic evidence including growth performance, tissue histology and biochemical profiles. Nevertheless, direct quantitative measurements of energy budget parameters, including voluntary feeding rate, respiratory oxygen consumption and excretion efficiency, were not performed in the current work. Further research employing direct physiological calorimetry and quantitative energy flow analysis will be necessary to validate the interpretive framework proposed here and clarify the underlying regulatory mechanisms.

Looking forward, more in-depth multi-scale studies addressing these gaps will provide a more robust theoretical foundation for the optimization of commercial aquaculture practices.

## 5. Conclusions

This study systematically compares physiological homeostasis and muscle quality of *L. vannamei* between open earthen ponds and RAS. The results demonstrate that the more controlled rearing conditions in RAS significantly improve growth performance and tissue health, while fluctuating pond environments induce multi-tissue pathological changes, causing energy to potentially shift from growth to stress compensation. These findings provide histological and physiological evidence supporting RAS as a strategy for high-quality shrimp production.

## Figures and Tables

**Table 1:** Growth performance of *L. vannamei* across culture systems.

Reference	Culture System	Stocking Density	Culture Duration	Control/Open System Outcomes	Treatment/Controlled System Outcomes
[10]	Biofloc-based tank system (indoor vs. outdoor)	580 ind/m^3^	13 weeks (91 d)	Outdoor biofloc: SR 81.3%;FCR 1.25; yield 7.36 kg/m^3^	Indoor biofloc: SR 82.7%; FCR 1.32; yield 6.75 kg/m^3^
[9]	Biofloc (BFT) vs. clear seawater (zero exchange)	312 ind/m^3^	30 d	Clear seawater (zero exchange): FBW 7.37 g; FCR 1.52	Biofloc system (BFT): FBW 8.25 g; FCR 1.08
[11]	Super-intensive ponds: biofloc (BFT) vs. water exchange	500 ind/m^2^	140 d	Water exchange pond (control): WG 3.02 g	Biofloc pond (BFT): WG 3.55 g; lower FCR
[15]	Intensive outdoor earthen pond (net cages)	450 shrimp/net	110 d	Outdoor earthen pond: IBW 1.06 g; FBW 20.05 g; FCR 1.36	—(single pond group)
[7]	Indoor recirculating aquaculture system (RAS), low salinity	500 PL/m^3^	84 d	—(single RAS group)	Indoor RAS: FBW 14.87 g; SGR 7.12%/d; FCR 1.32; SR 70%

Abbreviations: SR, survival rate; FCR, feed conversion ratio; FBW, final body weight; BFT, biofloc technology; WG, weight gain; IBW, initial body weight; SGR, specific growth rate; ind/m^3^, individuals/m^3^; PL/m^3^, Post-larvae/m^3^.

## Data Availability

The original contributions presented in this study are included in the article. Further inquiries can be directed to the corresponding author.
